# Enabling comparative modeling of closely related genomes: example genus *Brucella*

**DOI:** 10.1007/s13205-014-0202-4

**Published:** 2014-03-08

**Authors:** José P. Faria, Janaka N. Edirisinghe, James J. Davis, Terrence Disz, Anna Hausmann, Christopher S. Henry, Robert Olson, Ross A. Overbeek, Gordon D. Pusch, Maulik Shukla, Veronika Vonstein, Alice R. Wattam

**Affiliations:** 1 Mathematics and Computer Science Division, Argonne National Laboratory, Argonne, IL USA; 2 Fellowship for Interpretation of Genomes, Burr Ridge, IL USA; 3 Virginia Bioinformatics Institute, Virginia Tech, Blacksburg, VA USA; 4 IBB-Institute for Biotechnology and Bioengineering, Centre of Biological Engineering, University of Minho, Campus de Gualtar, 4710-057 Braga, Portugal; 5 Computation Institute, University of Chicago, Chicago, IL USA

## Abstract

For many scientific applications, it is highly desirable to be able to compare metabolic models of closely related genomes. In this short report, we attempt to raise awareness to the fact that taking annotated genomes from public repositories and using them for metabolic model reconstructions is far from being trivial due to annotation inconsistencies. We are proposing a protocol for comparative analysis of metabolic models on closely related genomes, using fifteen strains of genus *Brucella*, which contains pathogens of both humans and livestock. This study lead to the identification and subsequent correction of inconsistent annotations in the SEED database, as well as the identification of 31 biochemical reactions that are common to *Brucella*, which are not originally identified by automated metabolic reconstructions. We are currently implementing this protocol for improving automated annotations within the SEED database and these improvements have been propagated into PATRIC, Model-SEED, KBase and RAST. This method is an enabling step for the future creation of consistent annotation systems and high-quality model reconstructions that will support in predicting accurate phenotypes such as pathogenicity, media requirements or type of respiration.

**Electronic supplementary material:**

The online version of this article (doi:10.1007/s13205-014-0202-4) contains supplementary material, which is available to authorized users.

## Introduction

Since the first bacterial genome was sequenced in 1995 (Fleischmann et al. 1995), the number of genome sequences has grown exponentially (Lagesen et al. 2010). This increase in genomic data has demanded the improvements in high-throughput genome analysis tools that are widely being used today. It is now possible to automate the generation of annotations (Aziz et al. 2008) and initial draft metabolic models with minimal effort (Henry et al. 2010); however, the creation of accurate, high-quality models requires a substantial investment in mining phenotypic data (e.g., BioLog or RNAseq data) and an iterative reconciliation with the experimental data (Thiele and Palsson 2010).

The quality of the initial metabolic network reconstructions and their utility for formulating predictions depends on the quality and consistency of the annotations from which they were generated. If one attempts to compare the initial metabolic reconstructions for distinct organisms, a significant number of discrepancies in the resulting models are often found. However, isofunctional homologs must have the same annotations, so that they can be mapped to the same reactions in the models. Thus, improving annotation consistency and accuracy has become an issue of paramount importance.

In this report, we describe a broadly applicable protocol for improving the annotations and metabolic reconstructions for an entire genus. We demonstrate how this protocol has improved the annotations and metabolic reconstructions for genus *Brucella,* a group of intracellular facultative bacterial pathogens of humans and livestock. High-quality metabolic reconstructions and predictive metabolic models are available for several organisms, most notably model organisms such as *E. coli* (Orth et al. 2011) and *B. Subtilis* (Tanaka et al. 2013). A metabolic model for any *Brucella* strain has yet to be proposed. Since wet lab research with pathogenic organisms can be particularly challenging, this makes the development of predictive metabolic models for those organisms highly desirable. Maintaining annotation consistency among closely related genomes is the key step for enabling comparative modeling studies.

## Results

### Description of the protocol

*Step 1. Genomes are chosen for analysis* We have chosen fifteen genomes representing the major species, biovars and clades of the genus *Brucella* (Wattam et al. 2012) (Table 1).

**Table 1:** *Brucella* genomes used in this study with their SEED (Overbeek et al. 2005, 2013) and PATRIC (Gillespie et al. 2011; Wattam et al. 2013) identifiers, sizes, number of contigs and number of protein coding sequences (CDSs)

Genome name	PubSEED ID	PATRIC genome ID	Genome size (bp)	Number of contigs	Number of CDSs
*Brucella abortus* bv. 1 str. 9-941	262,698.4	15,061	3,286,445	2	3,413
*Brucella canis* ATCC 23365	483,179.4	25,663	3,312,769	2	3,394
*Brucella ceti* str. Cudo	595,497.3	28,239	3,389,269	7	3,578
*Brucella ceti* M13/05/1	520,460.3	83,544	3,337,230	22	3,367
*Brucella melitensis* bv. 1 str. 16 M	224,914.11	92,729	3,294,931	2	3,446
*Brucella microti* CCM 4915	568,815.3	92,249	3,294,931	2	3,374
*Brucella neotomae* 5K33	520,456.3	114,381	3,329,623	11	3,383
*Brucella ovis* ATCC 25840	444,178.3	136,990	3,275,590	2	3,499
*Brucella pinnipedialis* M292/94/1	520,462.3	74,143	3,373,519	15	3,356
*Brucella* sp. 83/13	520,449.3	75,385	3,153,851	20	3,152
*Brucella inopinata* BO1	470,735.4	109,945	3,366,774	55	3,361
*Brucella inopinata*-like BO2	693,750.4	146,994	3,305,941	174	3,276
*Brucella* sp. NVSL 07-0026	520,448.3	103,899	3,297,137	17	3,442
*Brucella suis* 1330	204,722.5	107,850	3,315,175	2	3,402
*Brucella suis* bv. 5 str. 513	520,489.3	73,489	3,323,676	19	3,316

*Step 2. Potential mobile element proteins are identified and removed from consideration* To find potential mobile element proteins we first identified repeat regions in each chromosome. BLASTN (Altschul et al. 1997) was used to compare each of the fifteen genomes against itself. Any DNA region (other than rRNA operons) occurring more than once in the genome with a nucleotide identity ≥90 % and a length ≥200 nucleotides was considered to be a repeat. Although there are many ways to identify mobile element proteins that could be substituted within this framework (e.g., Davis and Olsen 2011), for the purposes of this study, we define a potential mobile element protein as a one that overlaps a repeat region by at least 10 bp. All of the 15 *Brucella* genomes were then compared to the list of potential mobile element proteins using BLASTP, and matching proteins with ≥50 % identity over ≥80 % of the protein length were also considered to be potential mobile element proteins regardless of proximity to a repeat region. This resulted in the creation of 50 mobile element protein families, containing a total of 410 proteins. These proteins were excluded from subsequent steps due to their variability and because they are not currently used for metabolic model reconstructions.

*Step 3. Families of core proteins are generated* In order to find the core proteins, the remaining genes from each of the *Brucella* genomes were compared. Two proteins were placed in the same protein family if they were bi-directional best hits between a pair of genomes with >50 % identity and 80 % coverage, and the genes occurred within a conserved genomic context (Overbeek et al. 1999a, b). We considered the context of the matched pairs to be conserved if there were at least three pairs of bi-directional best hits co-occurring within a 10 Kb region. This resulted in 5,038 families (with two or more proteins) containing a total of 52,626 proteins. From these initial families we generated core protein families, which are defined as families containing at most one protein from each genome, where 80 % of the genomes are represented in the family. Similar to Step 2, it would be possible to substitute other methods for finding orthologous genes at this step as well (e.g., Li et al. 2003).

*Step 4. Annotation inconsistencies are removed* The core protein families of the RAST-annotated *Brucella* genomes were compared and inconsistencies (defined as two or more family members having different annotations) were evaluated. We manually curated a total of 398 families containing 4,848 proteins. We defined two metrics to measure progress.

The first:

*Given a protein family (i.e., from one of the 5,038 families we constructed), at what frequency has any given pair of proteins within the family been assigned precisely the same annotation by RAST (Overbeek et al.* 2013 *)?*

We report this property before and after manual cleanup, and compare our annotations to other public annotation resources (Table 2).

**Table 2:** The consistency of annotations across different resources

Source	Number of pairs	Number of pairs inconsistently annotated	Percent of pairs inconsistently annotated
RefSeq	562,597,217	383,808,122	68.2
IMG	101,525,838	52,434,525	51.6
TrEMBL	112,735,194	46,284,849	41.1
SwissProt	803,819	42,429	5.3
SEED	271,622,566	9,056,551	3.3
Original RAST output	16,349,603	102,097	0.6
RAST after manual curation	16,349,603	47,504	0.3

For each protein in a *Brucella* protein family used in this study, all of the proteins with identical sequences were found in various databases and the percentage of pairs that were inconsistently annotated was computed. Annotations were collected from RefSeq (Pruitt et al. 2007), UniProt Knowledgebase (UniProtKB)(Apweiler et al. 2010), the Translated EMBL Nucleotide Sequence Data Library (TrEMBL) (Boeckmann et al. 2003), the Integrated Microbial Genomes (IMG) system (Markowitz et al. 2012) and the SEED (Overbeek et al. 2005, 2013)

The second:

*How many Brucella*-*universal*-*reactions have been assigned to each genome?*

By universal reactions we mean the reactions that are present in all *Brucella* genomes used in this study. We chose this second metric to demonstrate that improvements in annotations lead to improvements in the metabolic reconstructions.

*Step 5. Annotation and reaction database improvements are made based on metabolic network reconstructions* Metabolic reconstructions were built for the fifteen *Brucella* genomes (Tables S1, S2), using the tools provided by DOE Systems Biology Knowledgebase (KBase) (http://kbase.us). Starting with the manually improved genomes, we focused on the reactions that were non-universal among the 15 *Brucella* strains. The annotations relating to these reactions were manually evaluated and corrected, if needed. This process was repeated.

The initial set of metabolic reconstructions from the original RAST annotations contained 1,011 *Brucella*-universal-reactions. The second set of reconstructions from the manually curated annotations (Step 4) contained 1,016, of which 20 were found to be new core reactions and 15 were removed from the set due to annotation errors. Finally, the third set, after using the metabolic reconstructions to guide the annotation cleanup, contained 1,047 *Brucella*-universal-reactions, of which 31 previously unrecognized core reactions were found.

### Annotation improvements

To eliminate sequencing, annotation and modeling errors from true strain-specific differences, we manually examined the 86 non-universal reactions from the second set of metabolic reconstructions. This revealed problems with the automated assertion or omission of reactions in certain genomes (Table S3). We verified the absence of 39 reactions from the set of genomes and identified 31 cases of *Brucella*-universal-reactions that had not been identified in the first round of metabolic reconstruction. The leading cause for the omission of reactions was insufficient sequencing quality (e.g., frame shifts, incomplete ORFs at the end of contigs or stretches of low quality sequence) that resulted in gene-calling errors. We also found 16 annotation errors (outdated functional roles), errors in the reaction database (labeled as “functional role ambiguities” in Table S3) and one gene fusion.

More importantly, this process resulted in the identification of five unique non-universal reactions in the *Brucella inopinata* BO1 and *Brucella inopinata*-like BO2 strains. Those reactions are involved in rhamnose-containing glycan synthesis and confirm the findings for those strains reported in (Wattam et al. 2012). In addition, we proposed candidate proteins in all Brucella for the *N*-acetyl-l,l-diaminopimelate deacetylase, the missing step in the diaminopimelate pathway (DAP) of leucine biosynthesis. All Brucella non-universal reactions for each genome are provided in Tables S4 and S5.

## Discussion

In this report, we have described a workflow for improving the annotations of an entire genus that utilizes metabolic reconstructions as a measure of annotation consistency. This has resulted in the production of an accurate and consistent collection of annotations and initial estimates of the metabolic network for the genus *Brucella*. By manual curation of 398 protein families (used in metabolic models) whose members had inconsistent annotations for isofunctional homologs, we have lowered the percentage of inconsistently annotated pairs of genes from 0.6 to 0.3 %. Those improvements have lead to changes in the metabolic reconstructions, generating a larger set of *Brucella*-universal-reactions and highlighting the real metabolic differences between organisms. We believe that knowledge of the real differences will be of importance when deciding on sets of “representative models” to portrait the entire genus. The “representative models” will aid in the research of less studied or newly sequenced strains.

With this work, we have demonstrated that the use of a controlled vocabulary for the annotation of genomes is a key for the construction of reaction networks and future predictive comparative models. The automated annotations provided by the RAST system and the SEED’s controlled vocabulary (Overbeek et al. 2005, 2013) provide a good start, but annotation inconsistencies caused by sequencing and propagation errors have to be manually processed. This method was devised to reduce the workload of researchers who are trying to build models, but it also clearly exposes bottlenecks where future computational tools must be built that can meet and exceed the skill level of an expert human annotator.

This work has improved the annotations in the SEED and RAST (Overbeek et al. 2005, 2013) and the reaction databases in Model-SEED (Henry et al. 2010) and KBase by flagging ambiguities in current functional roles. It has also improved the *Brucella*-specific collections of protein families that are propagated to RAST and PATRIC, the PathoSystems Resource Integration Center (Gillespie et al. 2011; Wattam et al. 2013), which is dedicated to enabling bioinformatics research for bacterial pathogens and has particularly strong ties to the *Brucella* research community.

With this proof of concept, we plan to use this methodology to improve annotations of other conserved genera and extend it to less conserved phylogenetic groups and pave the way for comparative modeling.

## Electronic supplementary material

Below is the link to the electronic supplementary material.

Supplementary material 1 (DOCX 22 kb)
