# Supplementary material for: Enabling comparative modeling of closely related genomes: example genus Brucella
Source: 3 Biotech. 2014 Mar 8;5(1):101–5. doi: 10.1007/s13205-014-0202-4 (PMC4327756; doi:10.1007/s13205-014-0202-4)
Supplement: Supplementary file 1 — Supplementary material 1 (DOCX 22 kb) [file 13205_2014_202_MOESM1_ESM.docx]

## Supplementary Material

The supplementary material is available via the PATRIC website at: http://enews.patricbrc.org/annotation_protocol_brucella/
